# Novel ELAC2 Mutations in Individuals Presenting with Variably Severe Neurological Disease in the Presence or Absence of Cardiomyopathy

**DOI:** 10.3390/life13020445

**Published:** 2023-02-04

**Authors:** Cérane Cafournet, Sofia Zanin, Anne Guimier, Marie Hully, Zahra Assouline, Giulia Barcia, Pascale de Lonlay, Julie Steffann, Arnold Munnich, Jean-Paul Bonnefont, Agnès Rötig, Benedetta Ruzzenente, Metodi D. Metodiev

**Affiliations:** 1 Laboratory for Genetics of Mitochondrial Disorders, Imagine Institute, Université Paris Cité, INSERM U1163, 75015 Paris, France; 2 Laboratory of Embryology and Genetics of Human Malformations, Imagine Institute, Université Paris Cité, INSERM U1163, 75015 Paris, France; 3 Genomic Medicine Service for Rare Diseases, APHP.CUP, Necker Enfants Malades Hospital, 75015 Paris, France; 4 Pediatric Neurology Department, Necker Enfants Malades Hospital, AP-HP, Institute Imagine, Université Paris Cité, INSERM U1163, 75015 Paris, France; 5 Department of Genetics, Reference Center for Mitochondrial Diseases (CARAMMEL), Necker Enfants Malades Hospital, 75015 Paris, France; 6 Reference Center for Inherited Metabolic Diseases, Necker Enfants Malades Hospital, Imagine Institute, Université Paris Cité, INEM-1151, G2M, MetabERN, 75015 Paris, France

**Keywords:** mitochondrial disease, ELAC2, RNA processing, cardiomyopathy, neurological disease

## Abstract

Transcription of mitochondrial DNA generates long polycistronic precursors whose nucleolytic cleavage yields the individual mtDNA-encoded transcripts. In most cases, this cleavage occurs at the 5′- and 3′-ends of tRNA sequences by the concerted action of RNAseP and RNaseZ/ELAC2 endonucleases, respectively. Variants in the *ELAC2* gene have been predominantly linked to severe to mild cardiomyopathy that, in its milder forms, is accompanied by variably severe neurological presentations. Here, we report five patients from three unrelated families. Four of the patients presented mild to moderate cardiomyopathy and one died at 1 year of age, one patient had no evidence of cardiomyopathy. The patients had variable neurological presentations that included intellectual disability, ataxia, refractory epilepsy, neuropathy and deafness. All patients carried previously unreported missense and nonsense variants. Enzymatic analyses showed multiple OXPHOS deficiencies in biopsies from two patients, whereas immunoblot analyses revealed a decreased abundance of ELAC2 in fibroblasts from three patients. Northern blot analysis revealed an accumulation of unprocessed mt-tRNA^Val^-precursor consistent with the role of ELAC2 in transcript processing. Our study expands the genetic spectrum of *ELAC2*-linked disease and suggests that cardiomyopathy is not an invariably present clinical hallmark of this pathology.

## 1. Introduction

Mitochondria produce energy for all eukaryotes utilizing a process called oxidative phosphorylation (OXPHOS) wherein the lateral transport of catabolic electrons is coupled with the generation of a proton gradient across the inner membrane that fuels the synthesis of ATP by the ATP synthase. This process is carried out by an identically named multienzyme system (OXPHOS system) composed of five multisubunit enzymatic complexes, complex I through IV (also known as the respiratory chain) and complex V, i.e., the ATP synthase. Four of these complexes (I and III–V) have a dual genetic origin containing subunits encoded by both the nuclear and mitochondrial genomes. In humans, the mitochondrial genome (mtDNA) encodes 2 rRNAs, 11 mRNAs and 22 tRNAs that are employed in the intramitochondrial synthesis of 13 polypeptides essential for the biogenesis of the dual-origin OXPHOS complexes [1]. Transcription of mtDNA starts from two opposing promoters and yields long polycistronic precursors containing the mitochondrial transcripts [2]. Within these precursors, in most cases, tRNA genes are interspersed between individual mRNAs and rRNAs. According to the so-called punctuation model for RNA processing, the tRNA locations serve as recognition sites for the nucleolytic release of the individual transcripts [3]. This is carried out by the RNAseP and RNAseZ nucleases, which cleave the RNA precursors at the 5′- and 3′-ends of the tRNAs, respectively, in a coordinated fashion. The RNAseP complex contains three subunits: MRPP1 (encoded by *TRMT10C*, Mendelian Inheritance in Man, MIM: 615423), MRPP2 (encoded by *HSD17B10* MIM: 300256) and MRPP3 (*KIAA0391*, MIM: 609947) [4]. MRPP3 is thought to carry the nucleolytic activity of RNAseP, which is strictly dependent on the presence of the other two subunits. MRPP1 and MRPP2 form a stable tRNA-binding complex that exerts a tRNA *N*1-methyltransferase activity via MRPP1, whereas MRPP2 alone is also a multifunctional enzyme involved in lipid and amino acid metabolism [5,6,7]. The only subunit of RNAseZ, ELAC2, is encoded by the *ELAC2* gene (MIM: 605367) [8,9,10]. This gene encodes two ELAC2 isoforms, the longer of which localizes to the mitochondrial matrix, while the shorter isoform, generated by an internal translation start site, localizes to the nucleus where it plays a role in the processing of nuclear-encoded tRNAs and miRNAs [9,11]. In mitochondria, both RNAseP and RNAseZ have been localized within the so-called RNA granules, which are sites of active RNA maturation [12]. Mutations in the genes encoding RNAseP and RNAseZ subunits have been linked to impaired precursor processing and tRNA maturation, OXPHOS deficiency and mitochondrial disease (MD) [13,14,15,16,17,18,19,20,21,22,23]. 

To date, twenty autosomal recessive variants in the *ELAC2* gene have been linked to MD, most commonly presenting with cardiomyopathy, developmental delay and lactic acidosis. Severe cardiomyopathy was usually associated with a poor prognosis in pediatric patients, while cases with milder forms had a prolonged survival, but variably severe neurological presentation [17,18,19,20,21,22,23]. Isolated complex I or combined complex I and IV deficiency were observed in patient biopsies and, sometimes, in fibroblast cultures consistent with the important role of ELAC2 in mtDNA expression. At molecular level, inactivation of ELAC2 led to accumulation of incompletely processed tRNA precursors (e.g., mt-tRNA^Val^-16S rRNA), occasional decrease in the abundance of some tRNAs but without any appreciable change in the abundance of mature rRNA and mRNA transcripts [5,17,21]. In a single case, it was demonstrated that an accumulation of tRNA precursors resulted in an inhibited mitochondrial translation and a decreased abundance of OXPHOS proteins in patient fibroblasts, suggesting a multiple OXPHOS deficiency in these cells [17].

Here, we present five patients from three unrelated families carrying previously unreported biallelic *ELAC2* variants. Clinical presentation of the patients was broadly consistent with the previously established clinical spectrum for *ELAC2* disease and included moderate or mild cardiomyopathy—in all but one case—neurological disease and developmental delay. We show that expression of these variants resulted in a decreased abundance of ELAC2 and an accumulation of mt-tRNA^Val^-16S rRNA precursor in patient fibroblasts. Our report expands the genetic spectrum of *ELAC2*-linked MDin humans and substantiates previous findings that *ELAC2* mutations do not always present with cardiomyopathy. Therefore, we propose that patients with neurological presentations, in the absence of cardiomyopathy, should also be suspected of carrying *ELAC2* mutations.

## 2. Materials and Methods

### 2.1. Exome Sequencing and Genetic Analyses

Sequencing was carried out in collaboration with the National Center of Genotyping (CNG). For patients (Pts) 1, 2, 4 and 5, genomic DNA was extracted from peripheral blood using the DNeasy Blood & Tissue kit (69504, Qiagen, Hilden, Germany) whereas, for Pt 3, genomic DNA was extracted from a muscle biopsy. Genomic DNA from Pts 1 and 4 was sequenced using a proprietary targeted next-generation sequencing panel including 215 nuclear genes linked to mitochondrial diseases (MitomeV1) and analyzed as described in [24]. Genomic DNA from Pt 5 was sequenced by whole exome sequencing as described in [25]. Sanger sequencing was used to identify the *ELAC2* variants in Pt 2 and Pt 3. In all cases, the pathogenicity of the identified missense variants was assessed in silico using the Sorting Intolerant From Tolerant (SIFT) [26] and PolyPhen 2 [27] algorithms included in the Alamut Visual 2.7 software (Interactive Biosoftware, Rouen, France).

### 2.2. Cell Culture

Control and patient skin fibroblasts were cultured at 37 °C in a humidified atmosphere with 5% CO_2_ in DMEM-GlutaMAX I medium (31966-047, Thermo Fischer Scientific, Montigny-le-Bretonneux, France) supplemented with 10% (*v*/*v*) FBS (10270106, Thermo Fischer Scientific), 100 U/μL penicillin, 100 μg/μL streptomycin (15070063, Thermo Fischer Scientific) and 50 μg/mL uridine (U3003-50G, Merck, Guyancourt, France). Cells were collected at ~90% confluence by trypsinization, washed with PBS and stored as pellets at −80 °C until needed. 

### 2.3. Whole-Cell Protein Extracts

Whole-cell protein extractions were carried out by suspending the cell pellets in RIPA buffer (89900, Thermo Fischer Scientific) supplemented with 1x Complete™ Protease Inhibitor Cocktail (11836145001, Merck) as described previously [28]. Following an incubation on ice for 20 min, the suspensions were sonicated and centrifuged for 20 min at 16,000× *g* in a cooled centrifuge. The supernatants were collected, frozen in liquid nitrogen and stored at −80 °C.

### 2.4. SDS-PAGE and Immunoblotting

The protein concentration of the cell lysates was determined using Bradford reagent (B6916-500ML, Merck). A total of 15–30 μg of whole-cell extracts, supplemented with Laemmli buffer (1610747, Bio-Rad, Marnes-la-Coquette, France) and 100 mM DTT (10708984001, Merck), were fractionated through precast 12% or 4–15% Criterion TGX polyacrylamide gels (5671044 and 5671084, respectively, Bio-Rad) and blotted onto PVDF membranes (1704275, Bio-Rad). OXPHOS proteins were fractionated through 12% gels and detected using the following antisera: anti-NDUFA13 (ab110240, Abcam, Cambridge, UK) for Complex I, anti-SDHA (ab14715, Abcam) for Complex II, anti-UQCRC2 (ab14745, Abcam) for Complex III, anti-cytochrome *c* oxidase (COXIV) (11242–1-AP, Proteintech, Manchester, UK) and anti-MTCO2 (COXII) (ab110258, Abcam) for Complex CIV, anti-ATP5A (ab14748, Abcam) and anti-ATP8 (26723-1-AP, Proteintech) for Complex V. For analysis of the abundance of ELAC2, LRPPRC and MRPP2, samples were fractionated through 4–15% gels and immunoblotting was carried out using the following antisera: anti-ELAC2 (10071-1-AP, Proteintech), anti-LRPPRC (sc-166178, SantaCruz Biotechnology, Heidelberg, Germany) and anti-MRPP2 (HPA001432, Sigma-Aldrich, Lyon, France). Anti-β-actin (20536-1-AP) and anti-α-tubulin (66031-1-Ig) were from Proteintech. IRDye-conjugated, secondary mouse or rabbit antisera were from Li-Cor Biosciences (926-32212 and 926-68073, respectively, Bad Homburg, Germany). Immunoblots were visualized with the Odyssey CLX infrared scanner (Li-Cor Biosciences) and quantified using Li-Cor’s Image Studio.

### 2.5. RNA Isolation and Northern Blot Analysis

RNA was isolated from control and patient fibroblasts using the miRNeasy kit (1038703, Qiagen) with on-column DNAse treatment. A total of 2 μg of RNA was fractionated through a 1.2% agarose gel and blotted onto a nylon membrane. Oligonucleotide (5′-TGGTCAGAGCGGTCAAGTTAAGTT-3′) complementary to mt-tRNA^Val^ was radiolabeled with 32P-γ-ATP using T4 polynucleotide kinase (M0201S, New England Biolabs, Ipswich, UK) and hybridized for overnight at 42 °C. Radioactive signals were detected by autoradiography using a BAS-IP Imaging plate (GE Healthcare, Tremblay-en-France, France).

### 2.6. Measurement of OXPHOS Activities

Enzymatic activities of individual OXPHOS complexes were measured spectrophotometrically as part of an established diagnostic routine at the Hôpital Necker-Enfants Malades, Paris, and as described in [29,30]. 

### 2.7. Statistical Analysis

Immunoblots were carried out on three independent samples from each cell line. Where applicable, one-way ANOVA was used for statistical analysis of the data. Analysis was performed in GraphPad Prism 9.4.1 (GraphPad Software, Boston, MA, USA).

## 3. Results

### 3.1. Case Reports

Patient 1, a boy, was the fourth child of first cousin Pakistani parents. He was born after a 38-week pregnancy and normal delivery. His birthweight was 3040 g, his height was 49.5 cm and his occipital frontal head circumference (OFC) was 33.5 cm. He first came to medical attention for transient, alternating myoclonic jerks of the inferior limbs, with strabismus and saccadic eye movements at 1 month of life. He developed feeding difficulties, frequent vomiting, gastro-esophageal reflux and laryngomalacia. His body growth curves gradually declined and, at five months of age, his head circumference was two standard deviations (SD) below the mean OFC for his age group (-2SD). He was hospitalized at 8 months for psychomotor regression with trunk hypotonia, peripheral hypertonia and cerebellar ataxia with distal limb tremor, varus deformity of the hindfoot and kyphosis. Routine workup revealed profound sensorineural deafness, optic disc pallor and mild hypertrophic cardiomyopathy. Brain magnetic resonance imaging (MRI) was initially normal but cochlear implant prohibited subsequent brain imaging. Electroencephalogram (EEG) was globally slow with neither spikes nor spike waves. At 4 years, he could not walk unaided due to spastic equinus deformity and cerebellar ataxia nor speak, but his oral comprehension was normal and he communicated in sign language. At 9 years, his condition was no longer progressing, and at 15 years old he was attending a school for children with special needs. At this age, he had sensorimotor neuropathy and a pharmacoresistant epilepsy with myoclonic jerks, tonic seizures and recurrent falls. Metabolic workup consistently revealed high plasma lactate from birth (plasma lactate: 3.6–10 mmol/L, normal range 0.9–1.8 mmol/L cerebrospinal fluid (CSF) lactate: 3.2 mmol/L, normal < 2.4 mmol/L) and urinary Krebs cycle intermediates. Spectrophotometric analysis of OXPHOS complex activities in muscle and liver biopsies revealed a decrease in the absolute activities of complexes I and III in muscle (Table 1). A younger brother, Pt 2, carried the variants detected in Pt 1. He also developed severe neurological disease with severe intellectual disability, sensorimotor neuropathy and cerebellar ataxia and, at the age of 13, he was wheelchair-bound. Like Pt 1, he developed pharmacoresistant epilepsy with myoclonic jerks and tonic–clonic seizures. He also had sensorineural deafness and mild cardiomyopathy.

Patient 3, a boy, was born to second cousin Malian parents after a 39-week pregnancy and normal delivery. Intrauterine growth retardation was noted during the first trimester and he was small for gestational age at birth (birthweight: 2550 g, height: 46 cm, OFC: 33 cm). He fed normally but shortness of breath and respiratory difficulties during feeds led to the detection of a heart murmur at one month of life. He failed to thrive, his body weight curve declined at 3 months, but his milestones of psychomotor development were normal. His condition gradually worsened at 4 months and a heart ultrasound detected a dilated and hypertrophic cardiomyopathy ascribed to severe subacute myocarditis (left ventricle posterior wall thickness: 35 mm, ejection fraction: 24%, shortening fraction: 11%). Pericardial effusion required urgent drainage at 4 months. Brain MRI and eye fundus were unremarkable. Metabolic workup showed normal plasma lactate and lactate/pyruvate molar ratios (lactate: 1.3 mmol/L, normal 0.9–1.8 mmol/L; pyruvate: 0.1 mmol/L, normal < 0.14 mmol/L; lactate to pyruvate ratio: 12, normal 6–14) but the accumulation of urinary lactate and Krebs cycle intermediates. At one year of age, he suddenly presented a fatal febrile circulatory shock in the context of a cough, rhinorrhea and lung infection. Spectrophotometric measurement of the OXPHOS activities in heart and liver biopsies from this patient revealed a multiple OXPHOS deficiency affecting complexes I, IV and V in both tissues and, in addition, a complex III deficiency in the liver (Table 1).

Patient 4, the younger brother of Pt 3, was born after a 40-week pregnancy and normal delivery. His birthweight was 3050 g, his height was 46 cm and his OFC was 33.5 cm. He did well during the first few months of life, but a systematic heart ultrasound at 4 months detected a moderate, concentric hypertrophic cardiomyopathy. He could sit unaided at 8 months and walk aged 17 months. At 2 years, he could say a few words, understand simple orders, eat and thrive normally (weight: 12.5 Kg; height: 89 cm; OFC: 47.5 cm). He had a mild gait ataxia but neither pyramidal syndrome nor cranial nerve involvement. The heart ultrasound showed a stable, well-tolerated hypertrophic cardiomyopathy with normal heart rate and no dyspnea. Metabolic work-up showed normal plasma lactate (2.3–2.7 mmol/L, normal 0.9–1.8 mmol/L). At the latest follow-up, his neurological examination was normal. He attended normal school but had difficulties reading.

Patient 5, a boy, was the first child of healthy first cousin parents from India. He was born after a full-term pregnancy and normal delivery. His birthweight was 3200 g, his height was 51 cm and his OFC was 33.5 cm. He had sucking and feeding difficulties with stridor, hypotonia, poor eye contact and several episodes of fainting, ascribed to vagal overactivity in the neonatal period. He required enteral nutrition during his first year of life. At 5 months, he had trunk hypotonia, limb hypertonia, strabismus, nystagmus and progressive microcephaly (OFC: 38 cm, −3.5 SD). His weight was 6700 g (−1 SD) and his height was 68 cm. He held his head at 10 months, could sit aided at 18 months and rolled over at 20 months. He had limb dystonia, speech delay and he required regular physical, occupational and speech therapy. No major dysmorphic features were noted. At last follow-up at 9 years, he had dystonia, limb spasticity and retractions requiring botulinum neurotoxin injections, but he could walk on tiptoes with aid. His weight was 20 Kg (−1.3 SD), his height was 116 cm (−1.5 SD) and his OFC was 47 cm (−4.3 SD). He was able to say words in three different languages but could not form sentences. He never experienced epileptic seizures. Eye examination showed oculomotor apraxia, bilateral convergent strabismus with lid ptosis and myopia. Extensive metabolic work-up, electromyography, EEG and heart ultrasound were all normal. 

All patients, except Pt 3, were alive at the time of writing of their respective case reports.

### 3.2. Genetic Studies

**Figure 1:**
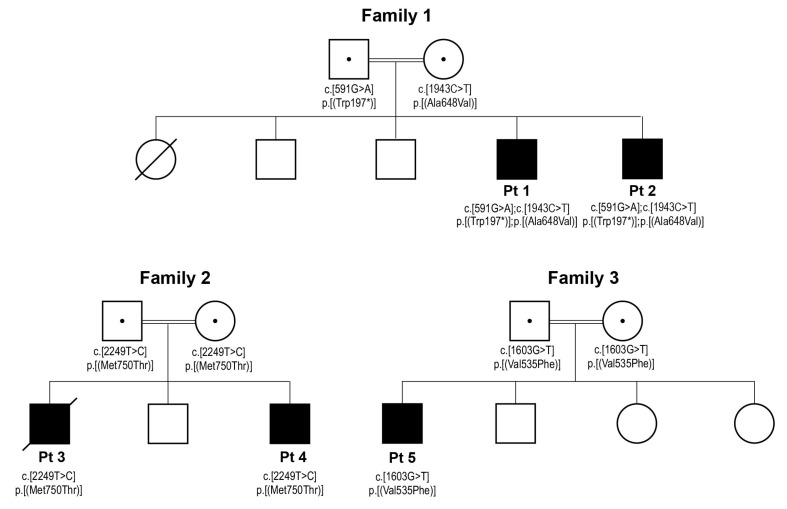
Segregation of the newly identified *ELAC2* variants in the three families. Standard pedigree and genetic nomenclature were used where squares and circles indicate male and female individuals, filled squares indicate male patients, empty squares and circles indicate healthy siblings, a diagonal strikethrough indicates a diseased individual, a central dot in the parental symbols indicates that they are carriers of the recessive variants, double horizontal lines connecting the parental symbols indicate consanguinity and “*” indicates a translation termination.

Targeted exome sequencing for Pts 1 and 2 revealed the presence of two compound heterozygous variants, a nonsense NM_018127.7:c.591G > A p.(Trp197 *) variant causing premature translation termination and a missense variant NM_018127.7:c.1943C > T p.(Ala648Val). Sanger sequencing and segregation analysis showed that the nonsense variant was inherited from the father, whereas the missense variant was inherited from the mother (Figure 1). For Pt 4, targeted exome sequencing identified a single missense variant NM_018127.7:c.2249T > C p.(Met750Thr). This variant was confirmed by Sanger sequencing in both proband’s parents and in a deceased sibling, Pt 3. For Pt 5, whole exome sequencing revealed a homozygous missense variant NM_018127.7:c.1603G > T p.(Val535Phe), which was confirmed in both parents by Sanger sequencing. 

**Figure 2:**
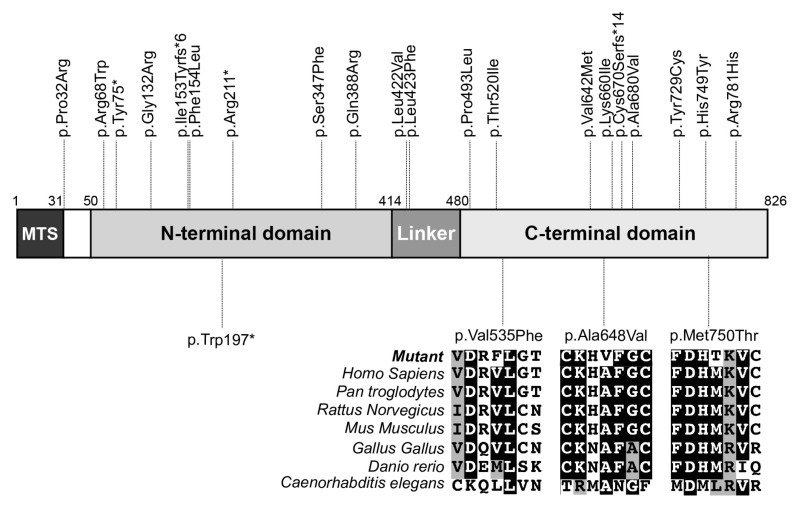
Map of all previously and herein-reported pathogenic variants along the ELAC2 polypeptide. Previously reported variants are shown on the top, while variants reported here are shown on the bottom part of the map. A multisequence alignment of the ELAC2 protein shows that all missense variants affect highly conserved amino acids. fs*N indicates a frameshift (fs) and a translation termination site (*) located N amino acids downstream of the frameshift site; MTS, mitochondrial targeting signal.

Next, we mapped the locations of all known disease-causing variants along the ELAC2 polypeptide, which revealed that they had a seemingly random distribution without any evidence of clustering in specific protein domains. A multiple sequence alignment with ELAC2 protein sequences from different species revealed that all missense variants affected highly conserved amino acids in the C-terminal domain of the protein (Figure 2). Finally, in silico analysis of the pathogenic potential of the missense variants suggested that all of them are potentially deleterious (Table 2).

### 3.3. Decreased ELAC2 Abundance and an Accumulation of mt-tRNA^Val^-16S rRNA Precursor in Patient Fibroblasts

**Figure 3:**
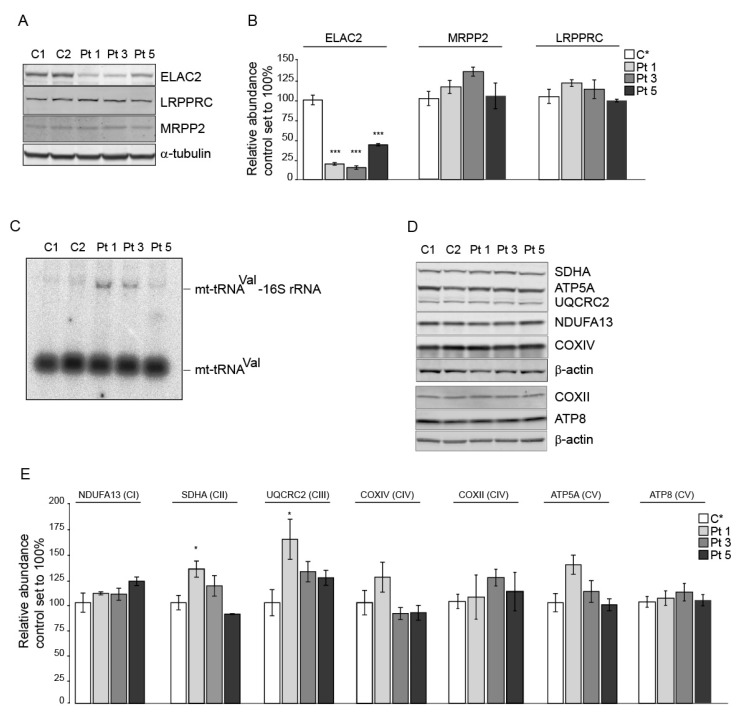
A specific and significant decrease in the abundance of ELAC2 and an accumulation of mt-tRNA^Val^-containing precursors in patient fibroblasts. (**A**) immunoblot analysis of control (C 1 and C 2) and patient (Pt 1, Pt 3 and Pt 5) fibroblasts demonstrating that the abundance of ELAC2 but not that of MRPP2 or LRPPRC is decreased in patient fibroblasts. (**A**) representative immunoblot is shown. α-tubulin was used as a loading control. (**B**) quantification of the immunoblots exemplified in A. Three independent samples from each cell line were analyzed. C*, averaged data for C 1 and C 2, *n* = 6; Pts 1, 3 and 5, *n* = 3; data are plotted as the average ± standard error. Statistics were one-way ANOVA, *** *p* < 0.001. (**C**) Northern blot analysis of RNA extracts from control and patient fibroblasts demonstrating the accumulation of mt-tRNA^Val^-16S rRNA precursor in patient cells. (**D**) immunoblot analysis of different OXPHOS proteins in control and patient fibroblasts. β-actin was used as a loading control. A representative immunoblot is shown. (**E**) quantification of the immunoblots exemplified in A. Three independent samples from each cell line were analyzed. CI to CV, OXPHOS complex I to V; C*, averaged data for C 1 and C 2, *n* = 6; Pts 1, 3 and 5, *n* = 3; data are plotted as the average ± standard error. Statistics were one-way ANOVA, * *p* < 0.05.

The detected variants affected highly conserved, potentially structurally important amino acids in the ELAC2 protein, which can affect its abundance in patient fibroblasts. Therefore, we carried out immunoblot analyses of fibroblast extracts from Pts 1, 3 and 5. These analyses revealed a statistically significant decrease in the abundance of ELAC2 in all patient fibroblast cell lines (Figure 3A,B). In contrast, the abundance of the RNAseP protein MRPP2, and that of the RNA stability and polyadenylation factor LRPPRC remained unchanged in these cells.

Because the inactivation of ELAC2 has been associated with an accumulation of mt-tRNA-mRNA/rRNA precursors, we proceeded to determine if, likewise, there was an accumulation of transcript precursors in fibroblasts from Pts 1, 3 and 5. Northern blot analysis using radiolabeled oligonucleotide complementary to mt-tRNA^Val^ revealed an accumulation of the mt-tRNA^Val^-16S rRNA precursor in Pt 1, Pt 3 and, much less so, in Pt 5 fibroblasts (Figure 3C). The latter also exhibited higher residual levels of ELAC2 protein, which could account for this result (Figure 3A,B). However, immunoblot analyses of nuclear- and mitochondrial-encoded OXPHOS proteins revealed no significant decrease of these proteins in Pt 3 and Pt 5 fibroblasts and a slight, likely compensatory, upregulation of some proteins in Pt 1 fibroblasts (Figure 3D,E).

Cumulatively, these data demonstrate the specific and deleterious effect of the identified ELAC2 variants leading to a decreased abundance of ELAC2 and impaired processing of precursor transcripts of mitochondria from patient fibroblasts.

## 4. Discussion

Here, we report the clinical presentations of five patients from three unrelated families expressing previously unreported variants in the *ELAC2* gene. Broadly, the clinical presentations reported here fit well within the previously established spectrum of *ELAC2*-linked disease. Patients carrying *ELAC2* variants commonly present cardiomyopathies that can be severe infantile cardiomyopathies—dilated and/or hypertrophic—often with a negative prognosis or milder cardiomyopathies, which are associated with prolonged survival. In the latter case, patients present a neurological phenotype, e.g., peripheral neuropathy [22], seizures [18], epilepsy [19] and sensorineural hearing loss [17]. In some cases, structural brain abnormalities affecting the cerebellum, brainstem and basal ganglia were documented [17,18,22]. In our report, Pts 1 and 4 had mild and moderate cardiomyopathy, whereas Pt 3 developed a severe dilated and hypertrophic cardiomyopathy and died at the age of one. Interestingly, Pt 5 did not have any evidence of cardiomyopathy on echocardiography. Only two other cases presenting without cardiomyopathy were previously reported, a case with developmental delay, constipation, microcephaly, ataxia and brain abnormalities [22] and a Korean patient with apnea, intractable epilepsy, growth retardation and developmental delay [19]. In the background of milder or absent cardiomyopathy, Pts 1, 2, 4 and 5 had variably severe neurological involvement including severe cerebellar ataxia, psychomotor regression, pharmacoresistant epilepsy, sensorimotor neuropathy, sensorineural deafness and optic pallor in Pts1 and 2; dystonia, spasticity and cerebellar ataxia with oculomotor apraxia, intellectual disability without psychomotor regression and ability to speak and walk with aid in Pt 5 and mild gait ataxia in Pt 4. Hypotonia, previously reported in *ELAC2* patients, was observed in Pts 1 and 5. Pts 1, 4 and 5 suffered varying degrees of developmental delay, which is commonly observed in long-surviving patients with *ELAC2* variants. Microcephaly, observed in Pt 5, was previously shown in several other patients including the one lacking cardiac involvement mentioned above [17,22]. Increased lactate was a frequent observation in *ELAC2* patients and was reported for Pt 1 while the other patients had normal (Pt 3, Pt 5) or borderline (Pt 4) lactate levels. Isolated complex I or multiple OXPHOS complex deficiency was reported frequently with *ELAC2* disease in tissue biopsies, which can be expected given the role of ELAC2 in the processing of mitochondrial transcripts. Diagnostic analyses of OXPHOS activities in biopsies from Pts 1 and 3 also revealed variably affected OXPHOS complexes. At least one study reported that *ELAC2* mutations cause a decreased mitochondrial translation and decreased abundance of OXPHOS proteins. However, our immunoblot analysis failed to detect any statistically significant decrease in individual OXPHOS proteins, arguing against a profoundly affected mitochondrial translation in these cells.

The small number of reported patients precludes the establishment of a firm phenotype–genotype correlation, as is the case for most nuclear genes linked to MDs. Known pathogenic *ELAC2* variants are distributed along the whole length of the ELAC2 polypeptide, and their specific position, zygosity or type (missense or nonsense) does not seem to correlate to a specific clinical presentation. It is also not clear to what extent individual mutations affect the expression, stability and activity of ELAC2 in different tissues. Recently, Saoura and colleagues examined the differential effect of individual variants on the activity of a purified ELAC2 enzyme against synthetic substrates in vitro [22]. While these studies did not address how the variants affect the stability of the individual mutant proteins, they clearly show that some mutations have a stronger inhibitory effect on ELAC2 than others, e.g., p.Pro493Leu and p.Tyr729Cys severely inhibited ELAC2 activity whereas p.Phe154Leu and p.Thr520Leu had a moderate effect on pre-tRNA processing. However, direct correlation between these data and the disease severity observed in patients expressing the variants in question cannot be inferred due to the in vitro nature of these studies. Interestingly, modeling of the p.Phe154Leu and p.Thr520Leu variants in Drosophila showed that, like in humans, expression of the Drosophila orthologs p.Phe155Leu and p.Thre494Ile results in cardiomyopathy [31]. It remains to be seen, however, if mutagenesis of any of the other amino acids in DmELAC2, which are orthologous to amino acids affected by disease-causing variants in humans, can likewise lead to cardiomyopathy or other pathology in Drosophila. The above-mentioned studies have undoubtedly set the stage for follow-up investigations that will help understand how different mutations lead to the clinical presentations ascribed to *ELAC2* disease.

In conclusion, our study expands the genetic spectrum of *ELAC2*-linked disease and suggests that cardiomyopathy is not an invariably present clinical hallmark of this pathology. Therefore, pathogenic *ELAC2* variants should be considered as a plausible cause of mitochondrial disease in patients with neurological presentations regardless of the presence of cardiomyopathy.

## Figures and Tables

**Table 1:** Diagnostic spectrophotometric measurements of the enzyme activities of individual OXPHOS complexes in biopsies from Pts 1 and 3. Enzyme activity in nmol/min/mg protein was measured spectrophotometrically. To calculate the relative activity for each complex, the absolute activity was normalized to the activity of citrate synthase (CS) when available. Bold typeface ↓, decreased activity; n.p., not performed.

		Patient 1				Patient 3			
		Muscle		Liver		Muscle		Heart	
		Pt	Control Range	Pt	Control Range	Pt	Control Range	Pt	Control Range
Absolute enzyme activity	CI	**32** **↓**	39–100	83.3	15–31.7	**4** **↓**	13–29	**21** **↓**	64–134
	CII	116	61–153	212.9	107–75	27	24–49	406	97–192
	CIII	**557** **↓**	714–1821	204.3	275–488	**204** **↓**	231–445	1575	1084–1872
	CIV	404	388–1237	260.6	130–237.4	**70** **↓**	111–252	**186** **↓**	459–863
	CV	372	202–506	347.8	59.7–128.8	**42** **↓**	55–128	**82** **↓**	139–327
Relative enzyme activity	CI/CS	n.p.	n.p.	1.01	0.26–0.51	**0.04** **↓**	0.14–0.22	**0.04** **↓**	0.22–0.32
	CII/CS	n.p.	n.p.	2.58	1.69–2.70	0.30	0.24–0.36	0.69	0.35–0.45
	CIII/CS	n.p.	n.p.	**2.48** **↓**	4.89–7.74	2.29~	2.30–3.30	**2.69** **↓**	3.20–4.27
	CIV/CS	n.p.	n.p.	3.16	2.27–3.81	**0.79** **↓**	1.26–1.98	**0.32** **↓**	1.51–2.10
	CV/CS	n.p.	n.p.	4.22	1.19–2.14	**0.47** **↓**	0.50–0.90	**0.14** **↓**	0.46–0.68

**Table 2:** Pathogenicity predictions for the identified *ELAC2* variants. Predictions were carried out in the Alamut Visual 2.7 software. Variants with a SIFT index < 0.5 (cut-off 0.05) are considered pathogenic. For Polyphen-2, a score closer to 1 indicates increasing probability that a variant is damaging. ^1^ accession number NM_018127.7; ^2^ accession number NP_060597.

Family	Patient	Variant ^1^	Amino Acid ^2^	Exon	SIFT (Score)	Polyphen (Score)
1	Pt 1, Pt 2	c.1943C > T	p.(Ala648Val)	21	deleterious (0.03)	probably damaging (0.998)
2	Pt 3, Pt 4	c.2249T > C	p.(Met750Thr)	23	deleterious (0.00)	probably damaging (0.996)
3	Pt 5	c.1603G > T	p.(Val535Phe)	17	deleterious (0.01)	probably damaging (0.979)

## Data Availability

The data presented in this study are available upon request from the corresponding author.
